# Supplementary material for: Bomb 137Cs in modern honey reveals a regional soil control on pollutant cycling by plants
Source: Nat Commun. 2021 Mar 29;12:1937. doi: 10.1038/s41467-021-22081-8 (PMC8007572; doi:10.1038/s41467-021-22081-8)
Supplement: Supplementary file 1 — Supplementary Information [file 41467_2021_22081_MOESM1_ESM.pdf]

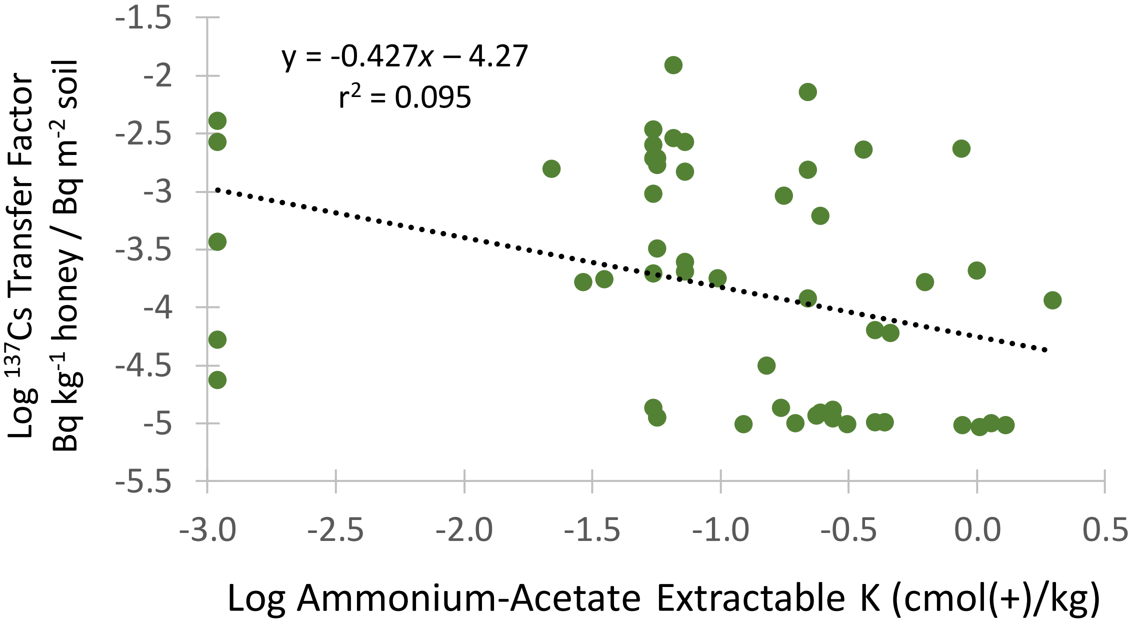

Supplementary Figure 1. The transfer of ^{137}Cs from soil to honey in the eastern United States scales inversely with ammonium-acetate extractable potassium (K) reported by the National Cooperative Soil Survey[30] (n=55). Extractable K is in units of centimoles (cmol) of charge (+) per kilogram (kg). We tested for the effect of extractable K on the ^{137}Cs transfer factor on log normalized data and provide linear function fit and r^2 adjusted for multiple comparisons ($p = 0.013$). This analysis only includes honey samples from counties for which the NCSS had data extractable K data for. Honey with undetectable ^{137}Cs are assigned half of the detection limit (0.015 Bq kg-1) for the transfer factor calculation and statistical testing.
