# Supplementary material for: Bomb 137Cs in modern honey reveals a regional soil control on pollutant cycling by plants
Source: Nat Commun. 2021 Mar 29;12:1937. doi: 10.1038/s41467-021-22081-8 (PMC8007572; doi:10.1038/s41467-021-22081-8)
Supplement: Supplementary file 4 — Description of Additional Supplementary Files [file 41467_2021_22081_MOESM4_ESM.docx]

Description of additional supplementary information

Title: Supplementary dataset 1

Description: All original data generated by this study are available as a supplemental dataset (Dataset 1) associated with the manuscript. All analytical results for <sup>137</sup>Cs and <sup>40</sup>K activities in the 122 honey samples are given along with 2-sigma analytical uncertainties in Dataset 1. Soil potassium concentrations and <sup>137<sup/>Cs deposition for each county is given, along with the standard deviation of the soil county potassium data.
