# Supplementary material for: Bomb 137Cs in modern honey reveals a regional soil control on pollutant cycling by plants
Source: Nat Commun. 2021 Mar 29;12:1937. doi: 10.1038/s41467-021-22081-8 (PMC8007572; doi:10.1038/s41467-021-22081-8)
Supplement: Supplementary file 5 — Reporting Summary [file 41467_2021_22081_MOESM5_ESM.pdf]

nature research

Corresponding author(s): James M. Kaste

Last updated by author(s): Feb 19, 2021

Reporting Summary

Nature Research wishes to improve the reproducibility of the work that we publish. This form provides structure for consistency and transparency in reporting. For further information on Nature Research policies, see our Editorial Policies and the Editorial Policy Checklist.

Statistics

For all statistical analyses, confirm that the following items are present in the figure legend, table legend, main text, or Methods section.

n/a	Confirmed	
☐	☒	The exact sample size (n) for each experimental group/condition, given as a discrete number and unit of measurement
☐	☒	A statement on whether measurements were taken from distinct samples or whether the same sample was measured repeatedly
☐	☒	The statistical test(s) used AND whether they are one- or two-sided<br>*Only common tests should be described solely by name; describe more complex techniques in the Methods section.*
☐	☒	A description of all covariates tested
☐	☒	A description of any assumptions or corrections, such as tests of normality and adjustment for multiple comparisons
☐	☒	A full description of the statistical parameters including central tendency (e.g. means) or other basic estimates (e.g. regression coefficient) AND variation (e.g. standard deviation) or associated estimates of uncertainty (e.g. confidence intervals)
☐	☒	For null hypothesis testing, the test statistic (e.g. F, t, r) with confidence intervals, effect sizes, degrees of freedom and P value noted<br>*Give P values as exact values whenever suitable.*
☒	☐	For Bayesian analysis, information on the choice of priors and Markov chain Monte Carlo settings
☒	☐	For hierarchical and complex designs, identification of the appropriate level for tests and full reporting of outcomes
☒	☐	Estimates of effect sizes (e.g. Cohen's d, Pearson's r), indicating how they were calculated

Our web collection on statistics for biologists contains articles on many of the points above.

Software and code

Policy information about availability of computer code

Data collection	Gamma spectrums were collected using Genie 2000 Software, version 3.2, Canberra Industries (2009). This was added to the methods section
Data analysis	Microsoft Excel for regressions, PAST 4.0, Paleontologia Electronica 4(10): 9pp. This information is now in the Methods section

For manuscripts utilizing custom algorithms or software that are central to the research but not yet described in published literature, software must be made available to editors and reviewers. We strongly encourage code deposition in a community repository (e.g. GitHub). See the Nature Research guidelines for submitting code & software for further information.

Data

Policy information about availability of data

All manuscripts must include a data availability statement. This statement should provide the following information, where applicable:

- Accession codes, unique identifiers, or web links for publicly available datasets
- A list of figures that have associated raw data
- A description of any restrictions on data availability

All original data measured in this study are available as a supplemental dataset associated with the manuscript. All analytical results for 137Cs and 40K activities in the 122 honey samples are given along with 2-sigma analytical uncertainties in Table S1. Soil potassium concentrations and 137Cs deposition for each county is given, along with the standard deviation of the soil county potassium data.

Field-specific reporting

Please select the one below that is the best fit for your research. If you are not sure, read the appropriate sections before making your selection.

☐ Life sciences ☐ Behavioural & social sciences ☒ Ecological, evolutionary & environmental sciences

For a reference copy of the document with all sections, see nature.com/documents/nr-reporting-summary-flat.pdf

Ecological, evolutionary & environmental sciences study design

All studies must disclose on these points even when the disclosure is negative.

Study description	We collected 122 unique honey samples from beekeepers in the U.S. and measured the nuclear contaminant Cs-137 alongside naturally occurring K-40 in each sample.
Research sample	The research sample is naturally produced honey, which tends to concentrate environmental contaminants. Samples were collected from producers who identified it as raw, pure, and unaltered. The honey samples were opportunistically collected to maximize a geographical extent and soil variability, along the eastern coast where atmospheric deposition rates where soil Cs-137 was constrained to approximately 1000 to 2000 Bq/m2.
Sampling strategy	Samples size was not pre-determined in a statistical fashion; rather we treated the study as a survey of Cs-137 contamination of honey in the eastern U.S. We chose an area that has approximately the same rainfall and distance from the test sites, but different geologic conditions. Our sample size gave us the power to show a significant regional trend in the Cs-137 incorporation into honey.
Data collection	Cs-137 and K-40 activities in the honey were determined by low-background gamma spectrometry using standard methods. Soil K data were extracted from a U.S Geological Dataset on soil chemistry derived from airborne radiometric surveys. J.Kaste and P.Volante performed the gamma analyses and A.Elmore extracted the spatial data on soil K.
Timing and spatial scale	The collection started in April 2017 and ended in March 2020. All samples were from North America but we focused our efforts on the eastern U.S. states where annual precipitation is approximately constant. A map is provided with all sampling locations that were analyzed for effects.
Data exclusions	We collected 122 honey samples and all were analyzed for Cs-137 and K-40. For 12 of the samples, we were uncertain of the pollinating area of the bees, so, we excluded these data from the spatial analysis of soil K.
Reproducibility	Our findings were not "experimemental", rather this study is a survey of Cs-137 contamination of honey that is available on the market. Samples were counted for at least 2 days using standard gamma spectrometry practices. Given the stability of intrinsic Germanium detectors, standard practice is to repeat a small number of analyses . We re-analyzed 5 samples and all efforts to reproduce the 137Cs and 40K data were successful.
Randomization	We analyzed samples from the northeastern U.S. and the southeastern U.S. on the detectors in a random fashion, alternating when we could.
Blinding	There was no group allocation in this study- soil K varied continuously across samples. However, all researchers were blinded to the sample source location, including source location soil potassium and source location soil 137Cs when analyzing the honey samples for 137Cs and 40K.

Did the study involve field work? ☐ Yes ☒ No

Reporting for specific materials, systems and methods

We require information from authors about some types of materials, experimental systems and methods used in many studies. Here, indicate whether each material, system or method listed is relevant to your study. If you are not sure if a list item applies to your research, read the appropriate section before selecting a response.

Materials & experimental systems

n/a	Involved in the study	
☒	☐	Antibodies
☒	☐	Eukaryotic cell lines
☒	☐	Palaeontology and archaeology
☒	☐	Animals and other organisms
☒	☐	Human research participants
☒	☐	Clinical data
☒	☐	Dual use research of concern

Methods

n/a	Involved in the study	
☒	☐	ChIP-seq
☒	☐	Flow cytometry
☒	☐	MRI-based neuroimaging
